# Harmonization of multi-scanner *in vivo* magnetic resonance spectroscopy: ENIGMA consortium task group considerations

**DOI:** 10.3389/fneur.2022.1045678

**Published:** 2023-01-04

**Authors:** Ashley D. Harris, Houshang Amiri, Mariana Bento, Ronald Cohen, Christopher R. K. Ching, Christina Cudalbu, Emily L. Dennis, Arne Doose, Stefan Ehrlich, Ivan I. Kirov, Ralf Mekle, Georg Oeltzschner, Eric Porges, Roberto Souza, Friederike I. Tam, Brian Taylor, Paul M. Thompson, Yann Quidé, Elisabeth A. Wilde, John Williamson, Alexander P. Lin, Brenda Bartnik-Olson

**Affiliations:** ^1^Department of Radiology, University of Calgary, Calgary, AB, Canada; ^2^Hotchkiss Brain Institute, Cumming School of Medicine, University of Calgary, Calgary, AB, Canada; ^3^Neuroscience Research Center, Institute of Neuropharmacology, Kerman University of Medical Sciences, Kerman, Iran; ^4^Department of Biomedical Engineering, Schulich School of Engineering, University of Calgary, Calgary, AB, Canada; ^5^Department of Clinical and Health Psychology, College of Public Health and Health Professions, University of Florida, Gainesville, FL, United States; ^6^Imaging Genetics Center, Mark and Mary Stevens Neuroimaging and Informatics Institute, Keck School of Medicine, Los Angeles, CA, United States; ^7^CIBM Center for Biomedical Imaging, Lausanne, Switzerland; ^8^Animal Imaging and Technology, Ecole Polytechnique Fédérale de Lausanne (EPFL), Lausanne, Switzerland; ^9^TBI and Concussion Center, Department of Neurology, University of Utah, Salt Lake City, UT, United States; ^10^Division of Psychological and Social Medicine and Developmental Neurosciences, Faculty of Medicine, Technische Universität Dresden, Dresden, Germany; ^11^Department of Radiology, Center for Advanced Imaging Innovation and Research, New York University Grossman School of Medicine, New York, NY, United States; ^12^Center for Stroke Research Berlin, Charité-Universitätsmedizin Berlin, Berlin, Germany; ^13^Russell H. Morgan Department of Radiology and Radiological Science, The Johns Hopkins University School of Medicine, Baltimore, MD, United States; ^14^Department of Electrical and Software Engineering, Schulich School of Engineering, University of Calgary, Calgary, AB, Canada; ^15^Division of Diagnostic Imaging, Department of Imaging Physics, The University of Texas MD Anderson Cancer Center, Houston, TX, United States; ^16^School of Psychology, University of New South Wales (UNSW), Sydney, NSW, Australia; ^17^Center for Clinical Spectroscopy, Department of Radiology, Brigham and Women's Hospital and Harvard Medical School, Boston, MA, United States; ^18^Department of Radiology, Loma Linda University Medical Center, Loma Linda, CA, United States

**Keywords:** magnetic resonance spectroscopy, harmonization, multi-site, multi-vendor, prospective, retrospective, brain

## Abstract

Magnetic resonance spectroscopy is a powerful, non-invasive, quantitative imaging technique that allows for the measurement of brain metabolites that has demonstrated utility in diagnosing and characterizing a broad range of neurological diseases. Its impact, however, has been limited due to small sample sizes and methodological variability in addition to intrinsic limitations of the method itself such as its sensitivity to motion. The lack of standardization from a data acquisition and data processing perspective makes it difficult to pool multiple studies and/or conduct multisite studies that are necessary for supporting clinically relevant findings. Based on the experience of the ENIGMA MRS work group and a review of the literature, this manuscript provides an overview of the current state of MRS data harmonization. Key factors that need to be taken into consideration when conducting both retrospective and prospective studies are described. These include (1) MRS acquisition issues such as pulse sequence, RF and B0 calibrations, echo time, and SNR; (2) data processing issues such as pre-processing steps, modeling, and quantitation; and (3) biological factors such as voxel location, age, sex, and pathology. Various approaches to MRS data harmonization are then described including meta-analysis, mega-analysis, linear modeling, ComBat and artificial intelligence approaches. The goal is to provide both novice and experienced readers with the necessary knowledge for conducting MRS data harmonization studies.

## Introduction

Magnetic resonance spectroscopy (MRS) is a non-invasive technique permitting detection and quantification of metabolites *in vivo*. Despite its potential to provide unique and useful diagnostic, prognostic, and biological information on healthy brain function and neurological and psychiatric disorders, MRS studies have generally been constrained by small sample sizes and poor reproducibility, limiting their interpretation and generalization. We suggest that this is one reason that MRS has not had the large-scale uptake of other MR research modalities such as functional magnetic resonance imaging (fMRI) or diffusion imaging. As MR research enters the information era, there is a growing need for big data analytics, robust pooling and harmonization of data across multiple sites. This paradigm shift motivated the establishment of The Enhancing NeuroImaging Genetics through Meta-Analysis (ENIGMA) consortium in 2009 with the initial goal of identifying replicable associations between common genetic variants and brain structure and function (1, 2). The successes of this initiative are widespread with over 50 working groups, including the MRS working group, with recent publications pooling imaging data from over 2,000 scientists from 45 countries to achieve the largest neuroimaging sample sizes for multiple disorders including schizophrenia [total *N* = 9,572; 4,474 cases; (3)], bipolar disorder [total *N* = 12,000; 3,500 cases; (4)], major depressive disorder [total *N* = 10,105; 2,148 cases; (5)], obsessive-compulsive disorder [total *N* = 3,665; 1,905 cases; (6)], and 22q11.2 deletion syndrome [22q11DS; total *N* = 944; 474 cases; (7); also see Thompson et al. (2)]. However, MRS research has not yet embraced data aggregation to this level and thus has not achieved studies with sample sizes of this magnitude. We believe this is largely due to difficulties in technical standardization and harmonization.

Multi-site studies offer an efficient way to increase sample size, which can be difficult at single sites due to a limited population for recruitment. Alternatively, when a single site has multiple scanners, it is possible that a single study will acquire data across multiple scanners for logistical reasons. Having multiple scanners at a single center also provides a controlled approach to investigate scanner effects as data from the same subjects, acquired by the same research team, can be acquired on multiple scanners relatively easily. A significant challenge with MRS is that even if there are two identical scanners, running the same software, operated by the same research team, there may be differences in their performance and ultimately metabolite quantification. Thus, for clarity, in this manuscript *multi-scanner* will indicate multiple individual scanners, which may be run at the same center, operated by the same team or at different locations. *Multi-vendor* will denote different scanner vendors (e.g., Canon, General Electric, Philips, and Siemens etc.) whether at the same location or multiple locations. *Multi-site* will refer to scanners at different locations that are operated by different teams and staff (so one institution may have multiple sites).

In this review, two approaches of data aggregation and multi-scanner studies will be considered: prospective and retrospective. Prospective development of multi-site/multi-vendor/multi-scanner MRS protocols should consider the technical factors and processing pipelines that will impact metabolite quantification. There are various approaches to this challenge, and each may be appropriate in different scenarios depending on the research question. One approach is to use the same scanner vendor and model, with the same software and the exact same parameters. However, this does not guarantee homogeneity of results as scanner characteristics such as eddy currents and frequency drift are dependent on the individual scanner (8). Further, it is likely not feasible to include only sites with identical scanners, as software upgrades are performed with inconsistent timelines. A lowest common denominator approach, where all sites use the same acquisition, catering to the least advanced scanner, regardless of the manufacturer, will minimize sequence variability. Even so, there are differences within the most conventional sequences between vendors limiting data homogeneity (9). This approach also results in underuse of some scanners' capabilities. An alternative is to take advantage of the most advanced capabilities on each scanner, acquiring data with the highest possible quality on each scanner and then combine results as effects rather than raw data. This approach carries the risk of not achieving the desired advantage of pooling multiple sites for increased power if the data quality is too diverse across scanners. Ultimately, a goal of harmonization is a balance between data consistency, data quality and data quantity.

There is a wealth of MRS data, published and unpublished, which may be aggregated using retrospective study designs to deepen our understanding of the brain and disease processes while leveraging the power of MRS data for future studies. With retrospective aggregation of data, there is little control over data acquisition parameters. While a scientist designing a study with retrospective data aggregation may choose inclusion and exclusion criteria in an effort to have highly homogeneous data, highly restrictive criteria may be self-defeating in that very few studies can be included in the analysis. As such, there is a need to consider how both technical and methodological differences will be handled. Furthermore, aggregating retrospective data requires biological factors to be considered as well.

**Figure 1:**
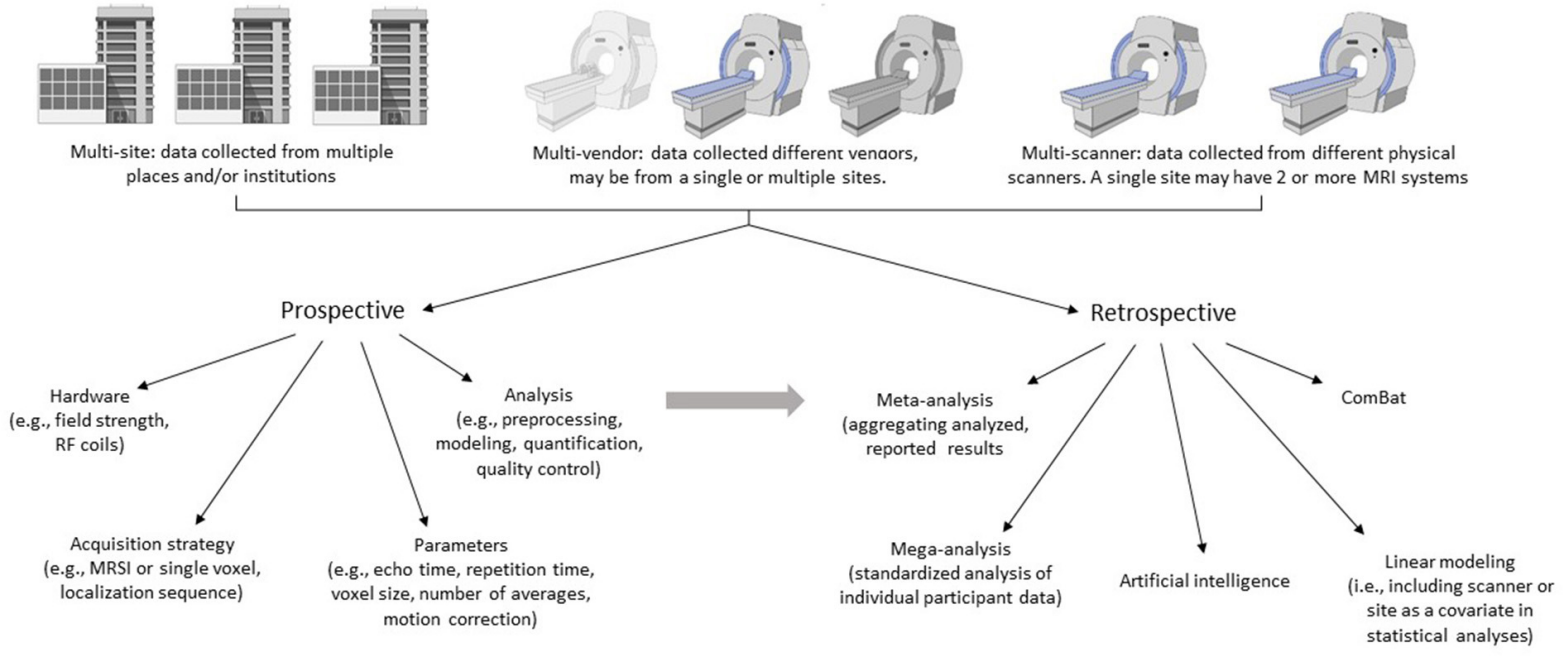
Aggregating multi-site, multi-vendor, or multi-scanner MRS data is an efficient way to increase sample sizes. Here we outline two approaches for data aggregation: Prospective and retrospective. Each may be appropriate in different scenarios depending on the research question and resources. The challenge and goal of aggregating data is to achieve balance between data consistency, data quality and data quantity. Prospective study designs should consider the technical factors and analysis pipelines that will impact metabolite quantification. Retrospective study designs aggregate previously collected data and therefore have little control over data acquisition parameters, instead focusing on analysis and harmonization strategies. The strategies applied in retrospective designs may also be applied to prospectively acquired data. Created with BioRender.com.

The goal of this review is to present the current state of MRS data harmonization including the unique technical/methodological and population/biological challenges associated with aggregating MRS data in both prospective and retrospective study designs (Figure 1). Considering the ENIGMA MRS working group's goal of developing data harmonization strategies, we will discuss several solutions employed to reduce these effects. Finally, we will present some interpretations and considerations related to these strategies and their application to study design in both prospective and retrospective multi-scanner, multi-site studies.

## Technical factors

Unlike MR imaging where quantitative imaging is more recent, MR spectroscopy studies have always been inherently quantitative. Initially, and still practiced, metabolites were quantified as a ratio to another metabolite (often creatine). This was followed by water referencing, and more recently, water referencing with appropriate corrections for water and metabolite parameters (T_1_, T_2_, and proton density) yielding absolute units based on the assumptions used (10, 11). One challenge with MRS quantification is that measurement variability depends on many factors, including hardware (scanner manufacturer, field strength, transmitter/receiver coils, passive shim elements impacting magnetic field inhomogeneities, and frequency drifts, etc.), acquisition parameters (localization method, echo time, repetition time, and voxel size, etc.), data quality signal to noise ratio (SNR), linewidth, and data analysis (pre-processing, fitting, and quantification). The next sections will overview how some of these factors impact harmonization of data between scanners, vendors and sites. For more in-depth background on MRS as a method, readers are directed to Oz et al. (12), Tomiyasu and Harada (13), Lin et al. (14), and Cecil (15).

### Hardware

Harmonization efforts may need to consider the available field strength and hardware. Different MRI scanners (even from the same vendor) have different RF coils used for transmit and receive. This is further complicated by many sites, particularly those at 7T, which use home-made RF coils. Additionally, the RF coil may impose additional limitations, such as maximum RF peak power. For prospective studies, scan protocols might have to be adjusted during study planning, so that they can be similarly executed on all types of participating hardware; however, this is challenging when aggregating data retrospectively.

### Acquisition

#### Localization

One of the first decisions in performing an MRS study is the localization approach. For single voxel spectroscopy (SVS), traditionally the choice was between STimulated Echo Acquisition Mode [STEAM, (16)] or Point RESolved Spectroscopy [PRESS, (17)], and more recently spin echo full intensity acquired localized [SPECIAL (18)], localization by adiabatic selective refocusing [LASER (19)], and semi adiabatic localization by adiabatic selective refocusing [semiLASER/sLASER (20)]. The selection of localization method has multiple consequences including possible limitations on echo time, differences in chemical shift displacement and SNR. SemiLASER is recommended in a recent consensus and is available for General Electric, Siemens and Philips scanners (21). Even so, it remains a research tool which cannot always be implemented at clinical sites with clinical systems due to limited experience with MRS or institutional regulations preventing the installation of research-based (e.g., works in progress) acquisitions. In addition, the use of an advanced sequence, such as semi-LASER, requires sites to obtain or generate basis sets for particular TE values, which can be difficult for novices. In contrast, STEAM and PRESS are available from all vendors as standard, product sequences. STEAM enables shorter echo times but has half the signal amplitude of PRESS (21). While PRESS and sLASER have comparable SNR at 3T, PRESS has on the order of ~12%/ppm chemical shift displacement effects, compared to sLASER which shows 2%/ppm (21, 22).

Magnetic resonance spectroscopic imaging (MRSI) combines MRS with spatial encoding methods to map spectral information over multiple voxels. The choice of acquisition methods (TE, volume selection/excitation scheme, and phase encoding scheme) will depend primarily on the study-specific spatial and spectral information requirements, while taking into account MRSI's limitations (23). PRESS and sLASER sequences are most widely implemented, with sLASER favored at higher field strengths owing to large chemical shift displacement errors with PRESS at ≥3T (20). The long acquisition times associated with MRSI has limited its incorporation into many clinical studies; however, there is a growing body of work focused on accelerating scan times. More advanced MRSI acquisition techniques using spatial-spectral encoding techniques with echo-planar readouts [EPSI (24); PEPSI (25)] or other non-Cartesian trajectories (26–28) are available. In addition, accelerated MRSI techniques using shorter repetition times or k-space under sampling [sensitivity encoding (SENSE), generalized auto-calibrating partially parallel acquisition (GRAPPA), multi-band/simultaneous multi-slice] have been developed [see Vidya Shankar et al. (29) and Bogner et al. (30) and references within]. These advancements remain largely in the research setting with limited implementation on clinical systems due to the need for specialized technical expertise to obtain good quality data. However, a recent consensus statement outlined vendor neutral recommendations for the acquisition, processing, and analysis of MRSI (23). As with SVS, even when the same parameters and acquisition approaches are used, substantial between-scanner differences can remain with MRSI data, this aggregating multi-site MRSI data will still need to account for differences between scanners (31).

#### Adjustments: RF calibration, shimming, water suppression calibration

There are multiple adjustments performed prior to the MRS acquisition that can have profound effects on data quality. These, in turn, may impact data harmonization. While many research centers use advanced methods to control these processes, many cannot be directly controlled by the operator. Thus, unless common vendors and systems are being used in a multi-scanner/multi-site study, the potential effects of differences in these adjustments may have to be accepted and their impact on data quality at a minimum recognized but likely controlled for in data analyses or aggregation steps. Each of these factors and their effect on data quality for harmonization will be briefly described here.

Radio frequency (RF) calibration is performed at the beginning of an exam and aims to optimize the RF power or RF voltage to yield the desired flip angle, typically with the aim of obtaining maximum signal (32). For SVS, this calibration is for the center of the voxel while the calibration is at the center of the volume of interest in MRSI. Performance of the RF calibration will ultimately affect sequence performance and data quality, particularly for PRESS localization. While most sites are not able to manipulate its efficacy, recognizing the impact on data quality may impact study design (i.e., choosing a single vendor) or data aggregation.

Linewidth directly impacts resolving overlapping resonances and complicated spectral patterns for reliable detection and quantification of metabolites. Narrow lines are a direct consequence of a homogeneous B**0** field, and in MRS, the required field homogeneity is generally higher than for most MR imaging applications resulting in an increased importance of shimming. Shimming generally refers to the process of (locally) removing macroscopic main magnetic field B0 inhomogeneities by applying small additional magnetic fields generated by shim coils. These are in addition to the passive shim elements that are also in place to develop a homogeneous main magnetic field and can impact field drift (described below). Often second-order shims are available on clinical systems, but this is not universal, with some systems having only first-order shims. Some research systems, particularly those at 7 T, have partial or full third-degree shims. While recommendations for SVS and MRSI shim approaches exist (33), customizing the shim methodology and hardware is generally not possible, particularly on clinical systems. Suggested criteria and corresponding linewidths have been defined for “excellent”, “adequate” and “acceptable” categories for multiple field strengths and various anatomical regions (33) which can be used as a practical approach to define thresholds for acceptable field homogeneity for data harmonization across scanners and sites.

Finally, if water suppression (WS) is applied, both the WS method and its calibration can impact data quality and thus its harmonization. The goal of WS is to yield an MR spectrum in which the water signal is smaller than, or about the same in amplitude as the largest metabolite peak [NAA in the healthy brain (34)]. Alternatives to WS have been proposed [e.g., metabolite cycling, (35)], though they are largely still in development. On clinical systems, water suppression enhanced through T_1_ effects (WET) with three or four CHemical Shift Selective (CHESS) WS pulses [WET, (36)], is the most commonly available WS method. A more elaborate method, VAriable Power and Optimized Relaxation Delays [VAPOR, (37)], uses a sequence of inversion pulses with varying inter-pulse delays and flip angles to minimize the water signal, although this approach is more available on research systems. Typically, the flip angle of a single WS pulse is varied during calibration to minimize the residual water signal amplitude. As with all pre-scan adjustments, differences in WS methods should be recognized as a contributing factor to vendor effects during harmonization, since systematic differences in residual water amplitude can affect the spectral modeling process.

#### Acquisition parameters

Accurate quantification of metabolite concentrations or ratios ideally requires long repetition times (TR > 2,000 ms for B_0_ ≤ 3T) and short echo times (TE < 30 ms) to minimize signal loss due to T_1_ and T_2_ relaxation effects (38). Of these, TE will have the largest impact on the number of metabolites detected and spectral quality. Short-TE spectra have higher SNR and facilitate the detection of metabolites that have short T2 relaxation times and/or J-modulation due to J-couplings [e.g., glutamate, glutamine, and myo-inositol (mI)]. However, shorter TE spectra are complicated by broad high molecular weight macromolecules (MM) (39). Analysis methods are available to separate metabolites from lipid/macromolecule signals (see below), which can be employed in processing pipelines when aggregating both prospective and retrospective data sets, but the overlap between metabolites and MMs (as well as between coupled metabolites) remains a substantial source of measurement uncertainty. In contrast, longer TE (135, 144, and 288 ms) acquisitions can be used to reduce macromolecule signals, due to their short T_2_ relaxation times. Optimizing the TE can also be used to optimize detection of a metabolite, for example, TE = 144 ms is used to improve the detection of lactate (40), which is inverted as a result of phase modulation by J-coupling. An alternative example is the recommendation of TE = 97 ms for the specific detection of 2-hydroxyglutarate using PRESS (41) or TE = 110 ms recommended for the detection of 2-hydroxyglutarate when using sLASER (42). While the choice of TE is typically determined by the study design (i.e., metabolite of interest) or the advantage of one technique over another (i.e., flatter baseline), the scanner gradient technology, and maximum peak B1 will constrain the minimum TE achievable on individual systems.

Two other important considerations are the voxel size and the number of averages (or number of transients) as these parameters both directly impact the SNR. When aggregating data from two studies that acquired different numbers of transients in the protocol, one approach may be to remove transients to match data acquisition parameters across scanners. However, deliberately reducing signal-to-noise and data quality is unsatisfactory, particularly if it impacts the net result (i.e., affects whether group differences are detected or not). Given the profound impact of voxel size on data quality, this is more challenging to harmonize directly. Alternatively, SNR and/or linewidth could be included as a covariate in analyses to mitigate the effects of data quality between sites or, in fact, between data sets. An additional confound of having different voxel sizes is the inclusion of different anatomy, which is discussed below. Other acquisition parameter selections such as number of spectral points and spectral bandwidth have less obvious effects on data harmonization procedures.

#### Motion correction

Motion is an issue with all MRS studies; first it degrades data quality by affecting frequency and phase. Motion also will degrade the shim and water suppression which further impacts data quality. Finally, motion will change the voxel localization, which may not actually degrade spectral quality, but data will be from a different location than expected (mislocalization) which will impact the interpretation of results. While it is not possible to determine whether differences in data quality are a result of motion or other acquisition factors, the impact of motion on data harmonization needs to be considered. At a base level, differences in immobilization techniques (*via* participant instructions or head restraints) or periodic B0 field changes resulting from respiratory or cardiac motion in individual participants across sites can contribute to differences in the extent of motion artifact between sites, an issue that may exist in both prospective and retrospective data harmonization. To mitigate the effects of motion, both retrospective and prospective correction methods exist, and a recent consensus paper recommends the use of an advanced motion correction that provides at least 5% stability or better (43). For prospectively designed studies, novel prospective motion correction approaches using internal navigation methods may be used. These approaches use additional pulse sequence elements to discern the position and shim parameters. Alternatively, external tracking systems that employ external cameras to continuously track motion combined with real-time B0 shim correction can minimize the impact of motion. We direct readers to Andronesi et al. (43) and references within for a review of the most advanced and effective motion correction methods. However, challenges with implementing such methods and ensuring comparable methods exist at all sites may prevail and, in those instances, guidelines for acceptable data quality should be based on the question to be addressed. A number of recent consensus papers outline key quality metrics [SNR, metabolite and unsuppressed water resonance linewidth, residual water signal, line shape, Cramér-Rao lower bounds (CRLB) of the line fit of the metabolites of interest, fit quality (residuals of the fit) and the presence of artifacts] to be evaluated when determining spectral quality (21, 38). There has also been work on automated ^1^H MRS quality control pipelines or combined approaches including both automated steps and visual spectra inspection as well as approaches with automated artifact pattern recognition for quality assessment (44–48).

### Analysis

Regardless of the acquisition parameters, common analyses are desired for harmonization. Even this can be challenging as there are multiple vendor-proprietary MRS data formats that export data in varying levels of “rawness”, e.g., all transients across all coils, coil combined, or in some cases, all transients are averaged prior to data export. The level of data rawness will impact both the degree of processing that can be applied as well as how common the analysis pipeline can be (for example, frequency-and-phase correction requires all transients), particularly in multi-vendor studies.

#### (Pre)Processing, modeling, and quantification

Modern MRS data analysis typically includes three key phases (11):

1) Data (pre-)processing, a series of operations preparing raw data into final spectra that are passed on to a modeling algorithm.
2) Modeling/fitting, the process of approximating/representing the measured signal.
3) Quantification, the process of converting model parameters into quantitative metabolite level estimates.

To accomplish these steps, a wide variety of data analysis software are available. While many widely used software packages such as LCModel (49) and jMRUI (50) have been distributed as compiled, closed-source binaries, there has been a notable shift toward modular open-source solutions (38, 51–53). LCModel, which has dominated MRS analysis, has also been made open access, but is not actively developed anymore. Many of these software packages have been compiled at https://www.mrshub.org.

#### Preprocessing

Recent MRS expert consensus has made several recommendations for preprocessing operations to maximize data quality, including receiver coil combination, alignment and removal of corrupted transients, eddy-current correction, etc., offering a template for harmonizing analysis workflows (11). However, there is no single comprehensive MRS data preprocessing pipeline that is universally agreed upon. As a result, MRS analysis workflows differ between research groups, and are frequently informed by personal preference and organic development over time, leading to incongruent results (54). While the effects of preprocessing pipeline diversity on metabolite estimates remain to be systematically investigated, it is difficult to imagine that this does not impact results and in turn affect the aggregation data across vendors at a minimum. Moreover, given the multitude of vendor-proprietary MRS file formats of varying levels of “rawness” developing a comprehensive MRS data preprocessing pipeline across vendors has so far proved elusive. Most recently, a standardized MRS-specific data storage format (“NIfTI-MRS”) and data/metadata accumulation logic (“BIDS-MRS”) have been proposed, which should mitigate pipeline heterogeneity, aid in large-scale data aggregation, and improve integration with other neuroimaging modalities (55, 56).

#### Modeling

The diversity of spectral modeling methods is another substantial source of discrepancy and variability in MRS. Recent expert consensus recommends linear-combination modeling (LCM) for most scenarios, i.e., approximating the measured data as the weighted sum of (measured or simulated) basis functions for metabolite and macromolecular signals, which is reflected by the majority of fitting algorithms available and used. Most LCM algorithms (11, 44, 49, 51–53, 57–61) include parameters for signal amplitudes, zero- and first-order phase adjustments and small frequency shifts. They differ considerably, however, in the way they estimate smooth baseline fluctuations and line shape parameters and employ different strategies to solve the (typically ill-posed) optimization problem, e.g., different ways to search for optimal starting values. The (perhaps unsurprising) resulting sensitivity of metabolite level estimates to small differences in the modeling process has long been informally considered an “open secret” in the MRS community, but is increasingly studied (62–64). These types of discrepancies can impact retrospective analyses, particularly when performing meta-analyses as data is not analyzed using a common process.

In some cases, harmonization of the modeling process across-site and within-site is possible assuming transparent reporting of as many details as possible (65). For example, to account for small differences between MRS sequence implementations in multi-site, multi-vendor studies, basis sets should be simulated as accurately as possible for each scanner, specifically incorporating the actual RF pulse waveforms, gradient schemes, sequence timing, etc. (9, 53, 62, 66–69). However, even with common acquisition parameters (as common as possible) and using vendor-specific basis sets, it should be understood that substantial variability remains between vendors and scanners (9).

An additional challenge in modeling is handling the baseline and macromolecules (MM). Expert recommendation is to include an experimentally measured macromolecular spectrum in the basis set, as mathematical approximation (by splines or polynomials, as implemented in most fitting software packages) “does not completely reproduce the *in-vivo* spectral pattern” (39) particularly at higher field strength (70, 71). However, measuring the macromolecules *in vivo* is technically challenging and time consuming thus often not feasible. Alternatively, MM can be (and often are) simulated based on the available parameters including frequency in ppm (usually to 0.9 ppm), linewidth, relative amplitude, number of components per resonance, etc. (39); however, this approach likely does not completely reproduce the *in vivo* spectral pattern. While this approach should also be useful for harmonization of multi-site studies (either prospective or retrospective), MM variance between scanners/vendors/sites is not well understood and still requires investigation.

#### Quantification

The final analysis step is the conversion of modeling parameters reflecting metabolite signal amplitudes into units representing concentrations. The most common approach is the use of an internal reference standard, i.e., a signal that is affected by the same proportionality factors including RF coil loading. For proton (^1^H) brain MRS, this standard is typically the internal water signal or the total creatine signal (creatine + phosphocreatine), though other references such as total NAA (NAA + NAAG) have been used. Using the tissue water signal as the reference requires an additional short measurement without water suppression. While many simply report this water-referenced quantification [reported as institutional units (i.u.)] it does not represent actual tissue concentration (i.e., true molar or molal units). Recommended procedures are provided in a recent expert consensus paper (11) to correct for relaxation and proton density in different tissues to approximate true, absolute concentrations. This consensus paper also underlines that long TR and short TE reduce the importance of relaxation correction, while acknowledging that their choice is often determined by the study goals. Measurement of individual relaxation times and tissue water content is prohibitively long in most clinical and research scenarios, and literature values are therefore most commonly used (72–76). The use of literature values complicates matters, as relaxation times and water content are field-dependent, and may vary in healthy and pathologic tissues (77–81). Quantification methods using external signals also exist but are beyond the scope of this text.

## Biological factors

Biological factors are crucial to consider in retrospective studies. In prospective study designs, effects of biology are generally considered *a priori* within the study designs, so are less of an issue for harmonization. For example, as metabolites and possibly macromolecules change with age, it is not possible to blindly combine data from different age ranges – this is also the case for many other imaging modalities. An additional challenge with MRS is that metabolite levels are linked to the tissue fraction in the voxel. Age-related atrophy therefore can impact metabolite levels and present as metabolite concentration decreases while in fact reflecting a decrease in tissue volume (82). Further, when considering the voxel and its anatomical location, metabolite levels vary throughout the brain with metabolite differences seen between white and gray matter and different lobes (83). This is further complicated by differences in water signal and signal relaxation (both metabolites and water) between these regions. While these factors can be prospectively accounted for, the ability to correct them retrospectively is limited to what level of raw data is aggregated. As with the above-mentioned technical factors, many of these factors are not independent and may further interact or be addressed methodologically in data aggregation and will be outlined below.

### Voxel placement and anatomy

The selection of anatomical targets (the voxel location) for a multisite study demands considerations beyond those of a single-scanner study. These considerations are compounded when using multiple platforms and vendors. While the scientific question at hand is paramount in selecting locations (i.e., prospective studies), voxel placement by those collecting data needs to be reliable and consistent. Typically, to ensure that data collection is consistent across sites, anatomical landmarks for voxel placement are recommended that are easy to identify on structural scans and require minimal anatomical sophistication among study staff. Common landmarks include midline, skull, genu of the corpus callosum and ventricles. As a guiding heuristic, manualized instructions that are pragmatic, with visual examples of each step involved in placement, should be provided to minimize judgments. Voxel rotations may present additional challenges, particularly across vendors that may differ in voxel manipulation flexibility, implementation and display of the voxel during placement and additional features such as the direction of the chemical shift displacement of the water signal (10, 38). This can result in the same procedure for voxel placement producing data collected in slightly different locations. While solutions to aid in reproducible placement of voxels across participants and sites such as SmartExam, AutoAlign (AA) scout, and AutoVOI (84) are available, they are not universally available and differ between vendors. Additionally, these tools need to be on all scanners to be useful for prospectively designed studies. Quantifying accuracy or voxel overlap between subjects can be difficult, as a voxel is generally of fixed volume, but intracranial volume (ICV) varies, so normalized voxels (e.g., using SPM, FSL, etc.) will produce voxels of varying sizes. This means that tools commonly used to quantify voxel overlap, e.g., the Sørensen-Dice Coefficient (85) or intensity images are limited in how high a value can be achieved, as they are evaluating overlap of areas that may no longer be the same size and are subject to the anatomical registration procedures used (i.e., selection of registration template and registration options such as linear, non-linear etc.). This does not mean that these tools should not be used, but rather to highlight their impact on interpretation.

### Age

The development of the brain lasts from birth until adulthood and brain metabolite concentrations are affected by normal development and aging. MRS studies have demonstrated developmental, age-dependent regional changes in NAA, creatine (Cr), choline (Cho), mI, glutamate/glutamine (Glx), and GABA (86–91) with the most significant changes seen in the first year of life (92–97). In neonates and infants, early rapid increases in NAA and Cr correspond to structural and functional development resulting from synaptic formation and increases in energy demand. An increase in glutamate, Glx, and GABA neurotransmitters' concentrations are observed early in development that reflect increase in neuronal activity and the demands of the glutamate-glutamine-GABA neurotransmitter cycles. In contrast, mI decreases rapidly within the first 3 months (94, 96) while Cho decreases gradually over time becoming stable within childhood (96, 98), corresponding to the ongoing myelination and white matter development (99). By adolescence and adulthood, most metabolites reach stable concentrations for a period before decreasing slowly with advancing age. In aging, reductions in neuronal density and activity, reduced mitochondrial activity, and decreased membrane synthesis, are reflected by lower regional levels of NAA, ATP, and phosphomonoester, respectively (100). Aging is also associated with lower glutamate concentrations (101). However, it is challenging to separate voxel composition effects from changes in metabolite tissue-concentration. For example, decreases in GABA levels with age are a result of atrophy/tissue loss and it is likely that the concentration of GABA in the remaining tissue is not actually decreased (82). Age-dependent changes in the T_2_ relaxation times - for example, those seen for NAA and Cr from adolescence with advancing age - will also impact metabolite level estimates (77, 80, 81). Small sample populations, the use of limited and/or partially overlapping age ranges, and the non-linear trajectories of metabolite changes over a lifespan (90, 96, 102) will prove challenging for harmonization. It will be essential that harmonization strategies evaluate the extent to which the treatment of age - either as a covariate or as a main variable of interest - is amenable to general linear models, or whether more sophisticated approaches are necessary.

### Sex

There is substantial evidence of sex differences in brain structure and function in adults, which may be present throughout the entire lifespan or emerge during development (103–107). In contrast, sex differences in metabolite levels are not clearly understood. Within the current literature, sex effects are divergent and conflicting, due in part to small sample sizes, differences in anatomical region and acquisition strategy, as well as the absence of absolute metabolite quantification.

In the adult population, both SVS and MRSI studies have reported sex differences in regional cortical gray matter and/or white matter for both NAA and myo-inositol (93, 108–113). However, other work shows no differences in metabolite levels between sexes in the healthy adult population (114–118). Similarly, sex differences in regional neurotransmitter concentrations have also been reported, with inconsistent findings (119, 120).

In the pediatric population, one study of children aged 6–15 years, reported no difference in metabolite levels (NAA, Cr, Cho, mI, lipid, and lactate) between males and females across multiple averaged regions including the frontal white matter, bilateral basal ganglia, bilateral hippocampi, and cerebellum (121). Similar results were also reported in a prior short-TE single-voxel PRESS study in children with cerebral palsy and normal development (122).

A number of studies also reported a significant interaction between age and sex, with differences in NAA/Cho during typical development and aging, with age-dependent alterations occurring earlier in males (123). Similarly, differences in NAA, Cr, and Cho levels (males < female) have been reported in the cingulate gyrus and supraventricular white matter in subjects aged 60–90 years using intermediate echo time (TE = 135 ms) MRSI (124–126). In addition, regional and sex variations in neurotransmitters appear to be affected by age, with faster rates of regional GABA decline in females (127) and faster rates of regional glutamate decline in males (128).

Given the considerable heterogeneity of metabolite concentration across all regions of the brain, which depend on tissue type (gray vs. white matter), subject age, and sex, it is imperative that comparisons between subjects or groups have comparative information for the same region, age, and sex, or at least covary for age and sex in the statistical analysis. Recent studies in neuropsychiatric disorders and diseases like Alzheimer's disease show substantial sex differences in prevalence and there are increasing reports of metabolic sex differences in these disorders that might also show in MRS studies (109, 129, 130).

### Pathologic condition

A major goal of harmonization efforts will be toward establishing whether a pathologic condition is associated with different metabolism compared to the non-affected (healthy) brain. Therefore, factors related to both the pathology-affected and control cohort must be considered. Given the inability to cover all neurological conditions, we can use traumatic brain injury (TBI) as an example where multiple injury severities and mechanisms of injury fall within the TBI spectrum. Perhaps unsurprisingly, metabolite differences are seen across the TBI spectrum and even within single categories. Moreover, these differences depend on other TBI-specific factors such as time-since-injury. Importantly, the definition of the control group may have substantial effects. For example, in mild TBI or concussion studies, non-head-injured controls are often described but this does not always exclude previous head-injury (either explicitly stated or not). Alternatively, to more specifically characterize the brain effects of concussion, orthopedic injury controls are increasingly being used as the control group. A recent study suggests that structural changes seen were not unique to concussion but common across concussion and orthopedic injury when comparing both to non-injured controls (131). Alternatively, local compared to whole brain differences are not always clear. Finally, in some conditions such as multiple sclerosis, or cancer, it is common to make measurements in the abnormal tissue and compare to a contralateral control region; however, the contralateral tissue may not be “normal” compared to a typical population either by the disease process or from treatment (i.e., chemotherapy, radiation therapy). Therefore, while this information is specific and relevant to individual studies, it will have an impact on appropriate use of data aggregation.

## Approaches for harmonization

The factors relevant for the harmonization of MRS data have been presented above. In some cases, these factors can be considered and accounted for, particularly in prospective designs. However, the fact remains that for MRS, differences between scanners exist and some level of harmonization is required when aggregating multisite data within the framework of prospective or retrospective multi-scanner study designs.

### Meta-analysis

Meta-analysis, which synthesizes summary statistics from previously published single site studies, has been used to aggregate and generalize MRS findings in an attempt to improve accuracy in drawing conclusions about a particular population. For example, while not comprehensive, meta-analyses of MRS data have been used to identify patterns of metabolite alterations in patients with multiple sclerosis (132), mild cognitive impairment, and Alzheimer's Disease (133), to understand variable results in traumatic brain injury (134) and evaluate the diagnostic potential of MRS in CNS tumors (135–137), characterize regional glutamate-glutamine and/or GABA in major depressive disorders (138, 139), schizophrenia (140), substance abuse (141), pain (142), and HIV (143, 144).

On the most basic level, in a meta-analysis effect sizes are aggregated and weighted by the sample size to provide an overall result across all studies included. Although meta-analyses are typically conducted in adherence with the Preferred Reporting Items for Systematic Reviews and Meta-analysis statement (PRISMA) (145), MRS data are affected by methodological heterogeneity (both technical and biological), variations in data quality, and the statistical approach reported in the original papers. Recently, Peek et al. (142) developed a quality assessment tool, MRS-Q, to assess the quality of MRS data included in meta-analyses but data was not excluded for studies not meeting all criteria, as is common with quality assessments within a meta-analysis. While data quality may impact results, excluding studies that meet *a priori* defined inclusion criteria will also create bias.

In addition to aggregating primary effects, analyses such as meta-regressions and moderator analyses can provide insight as to the relevance of additional factors of interest within each effect. For MRS, these additional approaches provide an opportunity to assess whether technical or biological factors influence the results. For example, in the recent meta-analysis by Joyce et al. (134) metabolite differences with TBI were affected by year of publication, age and voxel location (anatomy) indicating multiple technical and biological factors impact MRS results in TBI. Thus, MRS findings from a meta-analysis can do more than provide aggregated evidence of metabolite effects, they can provide new insight to factors that should be considered and controlled for in future multisite studies whether prospective or retrospective.

### Mega-analysis

The methodological diversity seen in meta-analysis can be minimized through the analysis of de-identified individual participant data (IPD) using an agreed-upon processing strategy. The use of IPD either as summary statistics or the raw data itself provides unique opportunities for data harmonization, defining processing pipelines and implementing a common analytical strategy. When raw IPD is accessible, single stage statistical mega-analyses can be performed by the coordinating facility. This is the design used in most ENIGMA studies with two key differences compared to traditional meta-analysis; (1) use of standardized analysis for all data which includes standardized quality control for data, and (2) use of random-effects models rather than fixed-effects to allow for variance across cohort, depending on the biology and demographics of the cohorts. This framework means that the effects across studies are considered to be from a distribution rather than simply weighting by sample size and the heterogeneity of effects can also be measured and reported. Statistical analyses using IPD offer several other advantages including improved consistency in inclusion criteria across sites, improved statistical power, and reduced biases (146). A number of comparison studies suggest more acceptable false positive rates and higher statistical power in mega-analysis with IPD when compared to meta-analyses (146, 147). While mega-analyses are an attractive alternative to meta-analyses to harmonize analysis methodology, specific challenges to MRS remain. As above, significant differences in data can exist based on differences in acquisitions. Furthermore, depending on the data exported by different sites, some components of the analysis pipeline may not be feasible, for example retrospective frequency-and-phase correction assumes availability of individual transients whereas some data formats only include an average spectrum. A related issue is fitting and metabolite quantification can be impacted by data quality, in particular metabolites with low concentrations may have biased results. To address this issue, one approach is to include SNR and/or linewidth as covariates in the analysis.

### Linear modeling

One of the most common approaches for raw data harmonization is to include the factors that need to be harmonized in the statistical model, for example controlling for site, vendor, age as covariates in an analysis of covariance model. The goal is to control for the variance introduced by each of these factors similarly as if they were effects of interest. The advantage of this harmonization approach is that it does not require additional tools and, in some cases, can be performed on summary data (i.e., individual metabolite levels) rather than requiring the raw data for reprocessing or IPD. While it also allows for the inclusion of multiple factors to harmonize such as site, vendor, field strength, age, sex etc. depending on the sample size, power limitation will exist and there must be sufficient sample sizes from each group (factor to harmonize across) as well as across all aggregated data. The disadvantage is a risk of collinearities, particularly in retrospective data aggregation (e.g., if different sites collected data in different voxel locations) and the risk of variance being partitioned in an unexpected manner resulting in incomplete or inefficient data harmonization.

A specific application of harmonization that uses linear regression is to harmonize white matter and gray matter across the whole brain data for MRSI data. An advantage of MRSI is the multi-voxel data can be leveraged to yield partial volume-corrected mean gray matter and white matter concentrations (148). Thus, it is reasonable to strive to integrate this considerable strength in efforts to harmonize MRSI data. Using various approaches based on linear regression or least-squares optimization, average gray matter and white matter concentrations per metabolite can be obtained (148) and these global gray matter or white matter metabolite concentrations may assist to harmonize data rather than these different tissues influencing results. The main caveat of the approach is that it assumes relative homogeneity of metabolite levels in the gray matter and white matter within the MRSI volume and, by extension, only changes that occur diffusely in either (or both) tissue types will be detected.

### ComBat

More recently, alternate harmonization procedures have been proposed in imaging and one that is gaining popularity is ComBat. ComBat is an empirical Bayesian method in which (a) factors to harmonize (e.g., sites) and (b) variance to preserve (e.g., biological group differences) are defined (149–151). Briefly, terms to preserve and to harmonize are defined in a multivariate linear mixed-effects regression. This linear model then estimates the mean and the variance for these terms. Using Bayesian methods, factors of interest along with model parameters are then estimated with the removal of effects from factors to harmonize. Removal of unwanted variance, such as that from site effects, is thought to improve power to detect differences of interest, for example biological differences between groups. ComBat was originally developed to harmonize gene expression data (149) but has shown efficacy in removing unwanted variance in MR data, specifically structural analyses of both diffusion tensor data (150), cortical thickness (151–153), and functional data (154). ComBat has recently shown promise for MRS data harmonization and it offers the advantage of maintaining meaningful quantification of metabolite levels (155). While ComBat is an increasingly common approach to harmonize MRI data, and is therefore expected to gain use in MRS, it is largely limited to the assumption that linear or quadratic models are sufficient to explain data variance, though extensions for higher order distributions are being developed.

### Artificial intelligence

Deep Learning (DL) has been used for brain MRI analysis to minimize the influence of the varying acquisition scanner/parameters, etc., known as data/domain shift or batch effects (156) and therefore AI holds unexplored potential for MRS data harmonization. DL models learn the relevant features to solve a problem from the raw data (i.e., MR images). Still, its black-box nature means it can easily pick up on nuances of the data that are not present across data points in multi-scanner, multi-vendor studies. In DL, Domain Adaptation (DA) strategies have been proposed for data harmonization, allowing the generalization of DL models across different datasets by avoiding domain specific decision making (157). DL models with consistent results across different imaging types, different groups of subjects (varying age ranges, sex, and pathology), and acquisition parameters are more reliable, allowing broader usage and facilitating the translation to clinical practice. DA strategies can unlearn not just batch effects but also other potential confounders in the data, such as sex and age (158), leading to domain-invariant features and consequent data harmonization (159).

Another common DA strategy is to use adversarial models (160) that can learn to transform samples into a harmonized reference space (161). While most current DL work on data harmonization in the medical imaging literature focuses on MR imaging, these DA strategies can be translated to MRS. The most significant challenges of deploying DA strategies in MRS are the necessity of having sufficient data, which often needs to be labeled to train these models, and hyper-parameter tuning of such models, which can be challenging and time-consuming (162). For retrospective studies, the data availability constraint is less critical, but for prospective studies, it is crucial. One potential solution to harmonize data prospectively is to leverage legacy data from past studies to initialize the corresponding DL models and use zero-shot learning DA techniques (163).

## Discussion/interpretations

Aggregating imaging data has provided great insight along with new directions of research. Given these successes, and the abundance of spectroscopy data available, aggregation of MRS data is a relatively unexplored area with great potential as there is every reason to expect similar advances can be achieved by aggregating spectroscopy data as seen with other neuroimaging modalities. However, there is a lack of established approaches for effective harmonization beyond the traditional meta-analysis approach used in systematic reviews. While valuable, meta-analysis approaches come with many limitations. For example, the impact of non-biological sources of variance (e.g., scanner, site, vendor, etc.) are generally not investigated, though recent work shows technical or non-biological factors effects can be identified (134) suggesting more sophisticated data harmonization is required to most accurately aggregate data for informative analyses.

One prospective approach is to minimize differences in acquisition between sites, which was successfully demonstrated by Deelchand et al. (164). However, there is broad acceptance in the field that technical differences exist between scanners in detail even when using nominally comparable acquisition approaches. This has been confirmed in recent multi-site/multi-scanner studies (9, 165) that aimed to mitigate site effects by using common acquisition parameters (to the extent possible) but still show substantial site effects. Given the significant site-effects reported in these studies, it remains unclear for prospective study designs whether the most suitable approach is to harmonize all acquisition parameters, selecting a lowest common denominator approach in terms of acquisition technologies, or whether each site should use their “best” methodologies available to have the most precise measures while accepting different sites will have different data quality and measurement precision. The solution to this challenge will be study-dependent and will also depend on factors such as the study objectives and cohorts being studied, the diversity of scanners and hardware, study duration and timeline including the potential for what upgrades are likely to occur during the study. We expect in most cases, investigators will opt for a hybrid approach (harmonize some hardware such as field strength and head coil but optimize acquisition parameters) aiming for a balance between maximum data quality while minimizing the risk of not being able to effectively combine data.

Beyond prospective harmonization, retrospective harmonization remains an area with little exploration for MRS. To our knowledge, the only work in this area compared ComBat and residualization to harmonize MRS data collected in controls across multiple sites and showed these methods are effective in removing non-biological sources of variance and may reveal biological factors with the augmented power achieved with large sample sizes that have been harmonized (155). The development and validation of effective retrospective harmonization procedures has multiple applications; first to augment the success of data aggregation from prospective study designs and to facilitate the aggregation of data collected through independent studies. There has been an increase in prospective multisite studies that include MRS (9, 166–173) which will likely benefit from additional data harmonization. Of great potential are the analyses and applications of aggregated data from previous studies, including mega-analyses of data from clinical groups, aggregation of normative data to best control for multi-scanner effects and aggregating data for artificial intelligence approaches. Included in this data aggregation is the possibility of developing fully automated, open access analysis pipelines that are cloud-based, or containerized, to facilitate consistent analyses across multiple users, scanners and sites.

In conclusion, MRS research, and by extension the clinical conditions being studied, has the potential to greatly benefit from data aggregation and harmonization. As outlined here, we define data harmonization to occur either prospectively at the time of study design or retrospectively, though retrospective approaches can be applied to prospectively acquired multi-scanner studies. For effective MRS data harmonization, we have defined two broad categories that impact data harmonization:

(1) Technical/methodological factors that consider the scanner, its hardware and software as well as acquisition and data processing.
(2) Biological factors that depend on the participants, such as their age, sex and anatomy being studied.

Overall, harmonization of MRS data facilitates the development of large sample sizes possibly yielding normative metabolite levels for different brain regions and various diseases that enables investigation of multiple interacting factors and the detection of subtle effects. This unlocked potential will further assist the transition of MRS from a predominantly research-based tool to clinical routine.

## Author contributions

All authors listed have made a substantial, direct, and intellectual contribution to the work and approved it for publication.
